# Alkali-labile gangliosides

**DOI:** 10.1007/s10719-023-10103-0

**Published:** 2023-01-25

**Authors:** Laura Mauri, Sandro Sonnino

**Affiliations:** Department of Medical Biotechnology and Translational Medicine, University of Milan, Segrate, Mi Italy

## Abstract

The structure and properties of a group of gangliosides modified by mild alkaline treatment are discussed. We will present the occurrence and the structure of gangliosides carrying the *N*-acetyneuraminic acid *O*-acetylated in position 9, the Neu5,9Ac_2_, and of gangliosides carrying a sialic acid that forms a lactone ring. Starting from biochemical data we will discuss the possible biochemical role played by these gangliosides in the processes of cell signaling and maintenance of brain functions.

## Introduction

Glycosphingolipids are a family comprising many structures differing in both the sugar content of the oligosaccharide chain and in the ceramide structure. Inside the glycosphingolipid family, gangliosides are characterized for containing one to six sialic acid units. The name “sialic acid” derives from the Greek word σίαλον (sìalon), saliva, that was found to contain it [1]. Sialic acid is a trivial name of all the derivatives of neuraminic acid, the 5-amino-3,5-dideoxy-D-*glycero*-D-*galacto*-non-2-ulopyranosonic acid. Sialic acid has been found in nature in many structures, but three are the most abundant, the *N*-acetyl-neuraminic acid, Neu5Ac, the *N*-glycolyl-neuraminic acid, Neu5Gc and the *N*-acetyl-9-*O*-acetyl-neuraminic acid, Neu5,9Ac_2_. Neu5Gc is not expressed in healthy humans. Sialic acid is a component of both glycoproteins and gangliosides, but only in the early 1950s, knowledge on gangliosides began to expand [2].

Gangliosides release sialic acid by acid pH. In contrast, ganglioside glycoside linkages are stable when treated with alkaline solutions. Nevertheless, analyzing the brain ganglioside composition of different species of mice by TLC, it was observed that some gangliosides disappeared using ammonia containing solvent systems [3]. *N*-acetyl-9-*O*-acetyl neuraminic acid, Neu5,9Ac_2_, is *O*-deacetylated under alkaline conditions and such a sialic acid was released from brain gangliosides by mild acid hydrolysis [4]. In-depth structural studies performed in collaboration with Roland Schauer (Christian-Albrechts-Universität, Kiel, West Germany) allowed to establish that mouse brain contains a GT1b with the external sialic acid of the disialosyl chain, *O*-acetylated at position 9 [5]. In the following years other *O*-acetylated gangliosides, including *O*-acetylated-GD1a and O-acetylated-GM3 in erythrocytes, *O*-acetylated-GQ1b in mouse brain and *O*-acetylated-GD3 in fetal rat brain and human melanoma, were characterized [6–8].

More complex was the identification of ganglioside lactones. The occurrence of ganglioside lactones was proposed in the past on the basis of ganglioside chemical properties, but they were not identified [9]. Studies on the existence of ganglioside lactones in nature was difficult due spontaneous ganglioside lactonization under precise mild acidic conditions. Nevertheless, data suggesting a relationship between human age and brain content of GD1b-lactone were in favor of this [10]. GD1b-lactone was also found in pig, rabbit, rat, mouse, and pigeon [10]. GM3-lactone was shown to occur in mullet milt [11] and GD2-lactone in neuroblastoma and glioma [12].

Then entering into the third millennium, papers reporting on alkali-labile gangliosides rapidly faded, probably due to difficulties to move from chemical to biochemical studies aimed to understand the role played by sialic acid *O*-acetylation and lactonization in determining specific aspects of cell biology.

## TLC identification of alkali-labile gangliosides

High-performance thin-layer chromatography (HPTLC) of gangliosides is a simple and low-costing procedure for the analysis of the ganglioside patterns. Thin-layer chromatography (TLC) plates and solvent systems are available for high resolution of the ganglioside patterns. The procedure does not allow the determination of the structure of the ganglioside but reference standards allow to speculate on structure and quantity. When the gangliosides are recognized by overlay with specific proteins, monoclonal antibodies or toxins, the structural characterization can be confirmed with almost absolute certainty. Thus the TLC of gangliosides can be the starting procedure to obtain information on the ganglioside pattern before undergoing mass spectrometry analyses. This latter analysis remains the most suitable system for ganglioside structural characterization.

In addition to this, TLC performed under specific conditions gives information on the presence of alkali labile gangliosides, both *O*-acetylated gangliosides and lactones. The procedure is applied to a non-previously alkali-treated ganglioside mixture and consists of a two-dimensional TLC on HPTLC silica gel plates, performed with the same non-alkaline solvent system for both runs. Prior to the second run the plate is exposed at room temperature for 5 h to ammonia vapors in order to split alkali-labile linkages. The vapors of ammonia release the *O*-acetyl groups from sialic acid yielding the alkali-stable parent ganglioside. Ammonia vapors work also on ganglioside lactones, but in this case two compounds are formed: the ganglioside amide and in lesser amounts the de-lactonized parent ganglioside. A solvent system like chloroform/methanol/0.2% aqueous CaCl_2_, 50/40/10 by vol., used for both runs of HPTLC, is capable of separating the majority of gangliosides [13] component of a complex mixture. The ratio among the three solvents can be modified to improve the ganglioside separation in the case of partial overlapping of gangliosides. At the end of chromatographic analysis, i.e. following the second HPTLC run, all the alkali-stable gangliosides appear lined along a diagonal starting from the origin; the spots corresponding to alkali-labile gangliosides lie outside of the diagonal and can be individually detected and quantified on the basis of their sialic acid content.

**Fig. 1:**
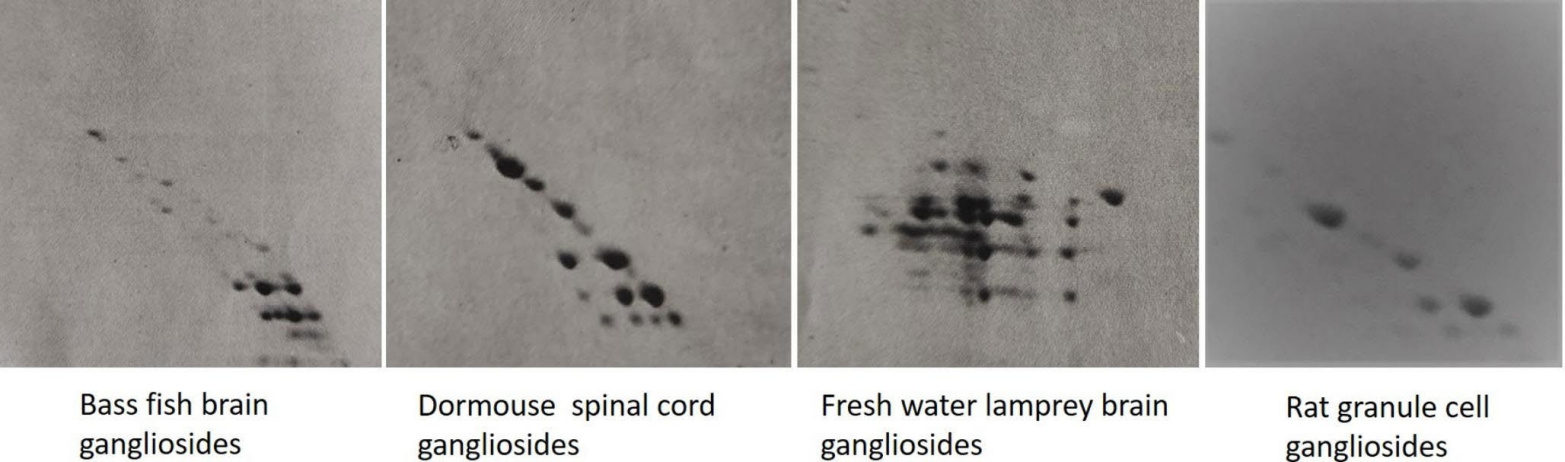
Two dimensional HPTLC of ganglioside mixture from brain bass fish, spinal cord dormouse, brain lamprey and cultured rat granule cells. Gangliosides were separated using chloroform/methanol/0.2% aqueous CaCl_2_, for both runs. Before the second run the plate was exposed to ammonia vapors for 5 h. Gangliosides were revealed using the paradimethylaminobenzaldehyde reagent. Different ratios of solvents were used to optimize ganglioside separation and Rf spots among the plates are not comparable. Polysialylated gangliosides with several acetylated sialic acids are aligned horizontally. The vertical alignment of some bands, with the higher one more intense, suggests the presence of lactones. Tissues were removed from the animals immediately after death and gangliosides rapidly extracted and analyzed. The HPTL is representative of a pool of animal tissues. The lamprey brain ganglioside mixture was unexpectedly comprising over 90% of alkali-labile gangliosides. Only one main spot was stable under ammonia vapors. The Rf of this spot overlaps with that of GD1a ganglioside

**Fig. 2:**
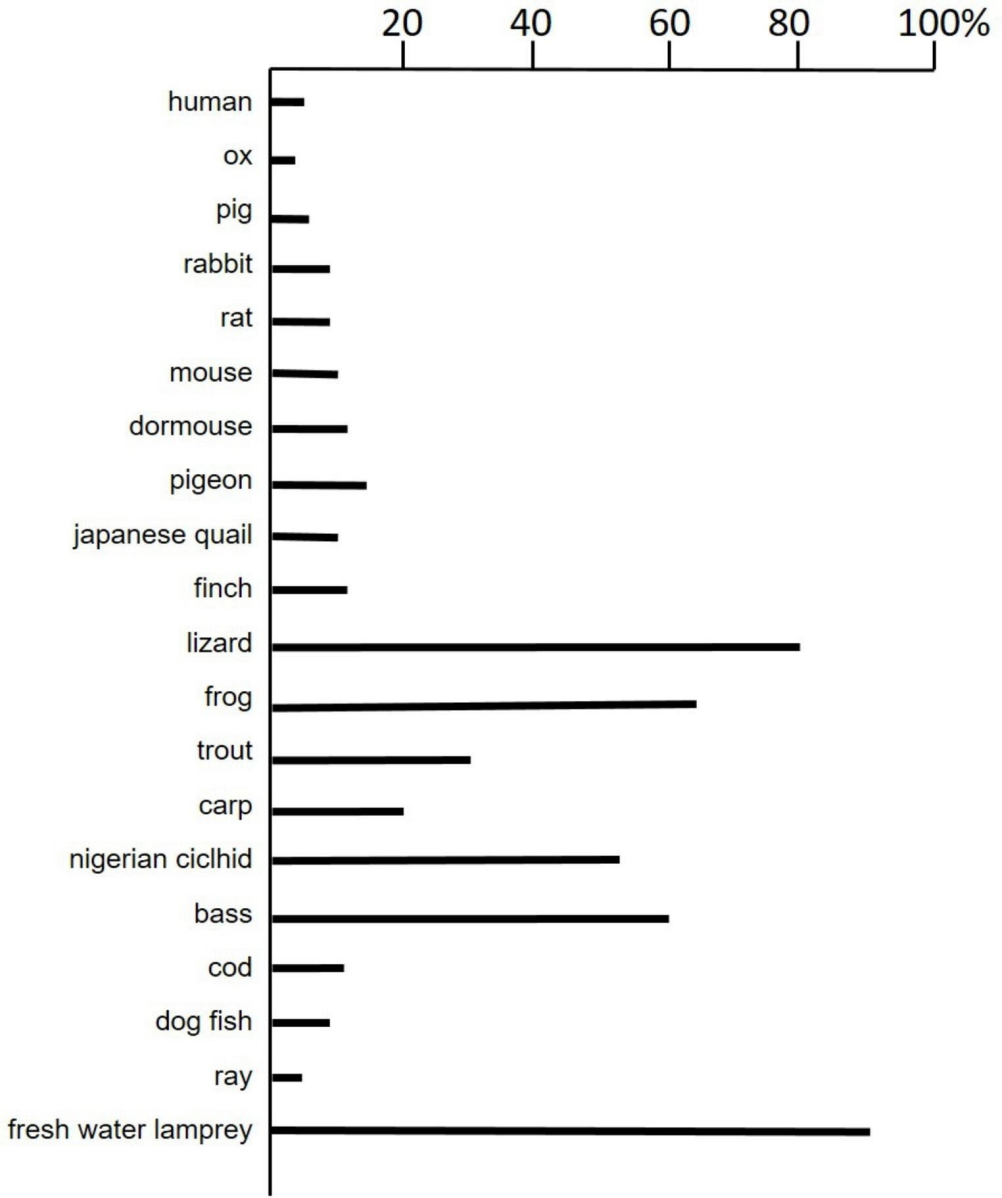
Alkali-labile ganglioside in the total brain of different species. Data are as % of sialic acid linked to alkali-labile gangliosides on the total ganglioside content. A detailed study on species grown up under different conditions has not been carried out. Values are indicative and for each species can vary due to subspecies, breeding conditions, environmental conditions, age and sex (data not shown and [8])

Gangliosides are particularly abundant in the nervous system and the content of alkali-labile compounds in the brain of several animal species has been determined. It varies from a few % to over 90%. Figure 1 shows a few examples of two-dimensional HPTLC of brain ganglioside mixture for the analysis of alkali-labile gangliosides and Fig. 2 compares the brain alkali-labile ganglioside content in several species. Two, three or more alkali-labile forms of a single core ganglioside were recognized. We have no information on the structure of these alkali-labile gangliosides. We can hypothesize multiple *O*-acetylation of a single core ganglioside.

The brains of fresh-water lamprey, reported in Fig. 2, were incredibly rich in alkali-labile gangliosides. The compound in only one main spot was stable under ammonia vapors. The Rf of this spot was overlapping with that of GD1a ganglioside.

## Gangliosides containing 9-*O*-acetyl-*N*-acetyl-neuraminic acid, Neu5,9Ac_2_

Sialic acid *O*-acetyl-transferase was reported to occur as a soluble cytosolic enzyme recognizing free sialic acid. It also occurs as a membrane enzyme acting on CMP-sialic acid [14]. No information is available on the kinetic properties of the enzymes for glycoproteins and gangliosides. More recent studies refer to a sole Golgi membrane enzyme and to the activity of acetyl-CoA acetyl transferase on sialoglycoconjugates [15]. The *O*-acetyltransferase transfers the acetyl group from acetyl-CoA to position 4, 7, 8 or 9 of sialic acid, both Neu5Ac and Neu5Gc. The acetyl group spontaneously shifts to position 9 from position 7 and 8, but not from position 4. The Golgi enzyme is a multi-membrane spanning protein showing high substrate specificity for CMP-sialic acid, suggesting that *O*-acetylation mainly occurs prior to the transfer of sialic acid by sialyltransferase. It is not clear if a family of enzymes exists, each enzyme being specific for the position of the sialic acid lateral chain, or if there is a single enzyme with broad position specificity. The enzyme activity is opposite to that of sialic acid esterase that removes *O*-acetyl groups from sialic acid. Thus, the final sialic acid structure is determined by a balance between the acetyl-transferase and the acetyl-esterase activities.

**Fig. 3:**
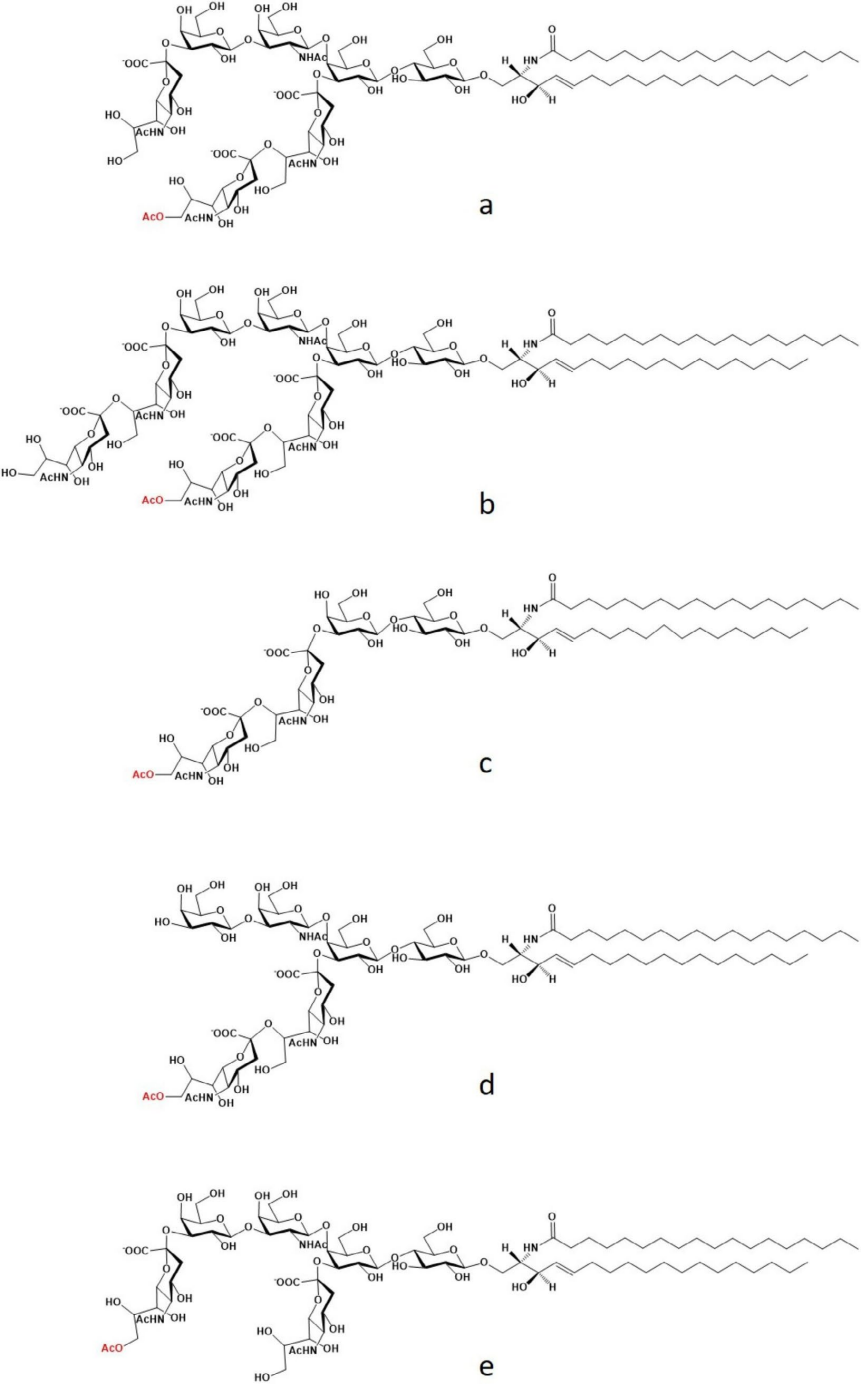
Structural representation of some alkali-labile gangliosides

The first precise information on Neu5,9Ac_2_ containing gangliosides goes back to 1977 [4]. Sialic acid was released under mild acid condition as well as by enzymatic treatment with sialidase from the total ganglioside mixture extracted from man, cow, horse, pig, sheep, cat, rabbit, rat, chicken and codfish, followed by mass spectrometry analyses. All the species contained Neu5,9Ac_2_ from a few % to up 20% of the total sialic acid content. The largest content of Neu5,9Ac_2_ was found in fish brains. In the following year [6] a ganglioside spot in the total ganglioside mixture from the brains of myelin-deficient mutant quaking mice was described to be alkali-labile. The ganglioside corresponding to the spot was isolated by a series of silica gel column chromatography and the final purified ganglioside was submitted to full characterization resulting a GT1b with Neu5,9Ac_2_ external to the disialosyl chain. This ganglioside according to official nomenclature is named IV^3^Neu5Ac-II^3^[(Neu5,9Ac_2_-(2–8)-Neu5Ac)]-Gg_4_Cer and its structure is reported in Fig. 3.

A few years later other *O*-acetylated gangliosides were characterized, such as the *O*-acetylated-GQ1b IV^3(^Neu5Ac)_2_-II^3^[(Neu5,9Ac_2_-(2–8)-Neu5Ac)]-Gg_4_Cer [6], the *O*-acetylated-GD1a IV^3^Neu5,9Ac_2_-II^3^Neu5Ac-Gg_4_Cer [7], the *O*-acetylated-GD3, II^3^[(Neu5,9Ac_2_-(2–8)-Neu5Ac)]-LacCer [8] the *O*-acetylated-GD2, II^3^[(Neu5,9Ac_2_-(2–8)-Neu5Ac)]-Gg_3_Cer [16] and the O-acetylated-GM3 II^3^Neu5,9Ac_2_-LacCer [11] (see Fig. 3 for some structures).

In spite of the progressive information on the presence of *O*-acetylated gangliosides in many mammalian species, and on their abundance in the brain of fish and other species, the information on the natural meaning of these alkali-labile compounds displaying higher hydrophobicity with respect to their parent gangliosides remains obscure. The main information on the biochemical properties of gangliosides containing O-acetylated-sialic acid is their partial resistance to sialidase activity. This could be important if we consider that *O*-acetylated-gangliosides seem to be associated with neuronal development, functions, and proliferation processes.

A specific role of *O*-acetylated gangliosides in nervous system functions is suggested by studies on normothermic and hibernating dormice. The brain of adult active dormice (*Glis glis*), maintained at 22 °C, is rich in *O*-acetylated gangliosides. They are components of olfactory bulb, forebrain cortex, midbrain, cerebellum, brain stem, pons and spinal cord, from a minimum of 10.2% in olfactory bulb, to a maximum of 30.1% in spinal cord, values mainly due to *O*-acetylated-GT1b and *O*-acetylated-GQ1b in all regions. These gangliosides disappeared in the main regions of the brains of hibernating animals (maintained at 6 °C), being only present in scant amount in brain stem and olfactory bulb [17]. This suggests a specific role of *O*-acetylated gangliosides in maintaining and regulating neuronal functions.

This seems confirmed by the behavior of rabbit brain *O*-acetylated-GT1b and *O*-acetylated-GQ1b that, particularly in cerebellum, progressively increased as single gangliosides and as % of total alkali-stable compounds, from birth to 6 months of post-natal life [18].

*O*-acetylated-GD3 was found in both normal and tumor tissues. In the rat nervous system, the *O-*acetylated-GD3 is present in discrete quantity during the latter part of embryonic development and the early postnatal period [19]. It has been proposed that *O*-acetylated-GD3 has a specific role as anti-apoptotic ganglioside, a role opposite to that of the apoptotic GD3 [20]. During the early stages of human development, *O-*acetylated GD3 is present in different tissues. In contrast, in erythropoiesis its level is decreased during maturation in the erythroid progenitor cells in the bone marrow. Enhanced presence of *O-*acetylated-GD3 has been reported in many cancer tissues, such as in breast cancer, basalioma, tumors of neuroectodermal origin, childhood lymphoblastic leukemia (ALL), and glioblastoma [21]. In some patients only metastatic lesions expressed the *O-*acetylated-GD3 whereas the primary tumor expressed exclusively the non-*O-*acetylated ganglioside [22].

## Ganglioside lactones

**Fig. 4:**
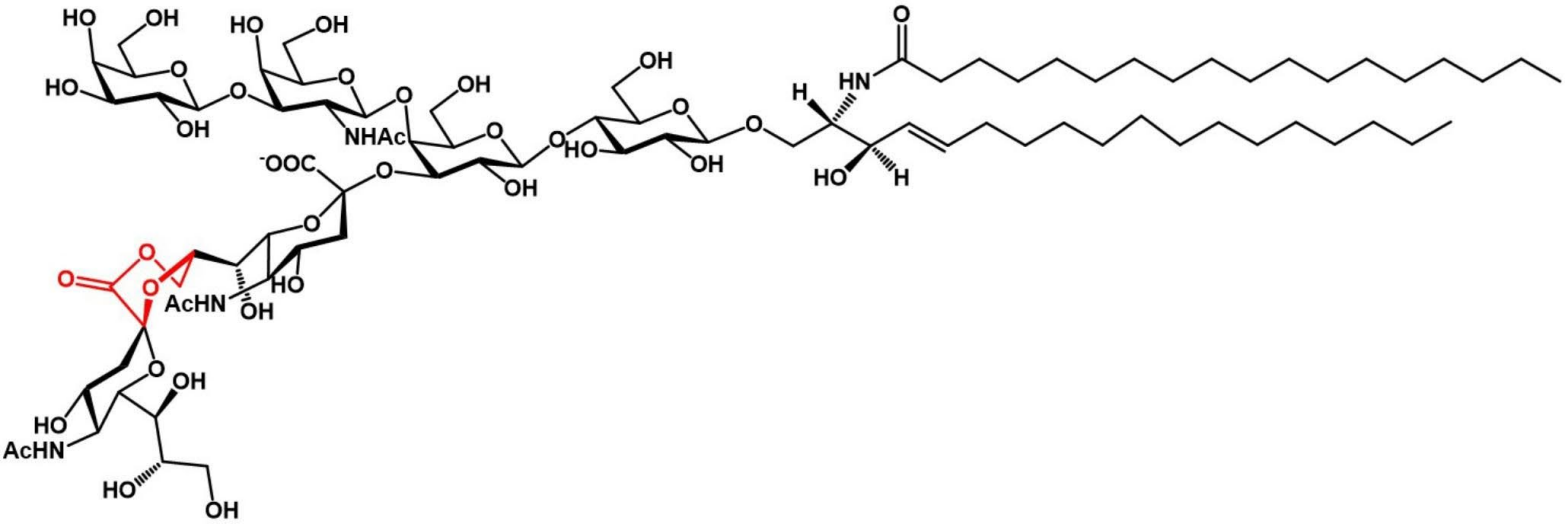
Structure of ganglioside GD1b-lactone isolated from human brain

On the basis of ganglioside chemical properties, the existence of ganglioside lactones was proposed in the 60s [9] and suggested later to occur in the ganglioside mixture from bovine adrenal glands [23]. These lactones have been proposed to occur in the ganglioside mixture from rodent brains following sodium borohydride reduction [24]. Finally, in 1987 a ganglioside lactone was isolated from human brains and was identified by structural characterization as the monolactone derivative of GD1b, with the inner ester within the disialosyl chain and the external carboxyl group and the hydroxyl group at position 9 of the lateral chain of the internal residue. The ganglioside was named GD1b-lactone and according to the official nomenclature has the structure II^3^[(Neu5Ac-(2–8,1–9)-Neu5Ac)]-Gg_4_Cer, shown in Fig. 4.

The pKa of sialic acid is 2.2–2.5 and gangliosides present negative charges at the cell surface. This allows to attract positive ions and water and to give ionic linkages with neighboring molecules. This may be responsible for the multiple functions played by gangliosides to modulate membrane receptor activity and cell signaling [25].

Two processes can be involved to modify the ganglioside membrane electronegativity. One process is catalyzed by the two membrane enzymes sialyltransferase [26] and sialidase Neu3 [27] that control the number of sialic acid residues. The second process involves the formation of inner ester linkage between the sialic acid carboxyl group and one of the hydroxyl groups in the same ganglioside molecule, yielding a ganglioside lactone. This latter process, in addition to rendering the oligosaccharide chain less negative, it makes the ganglioside resistant to sialidase, preventing the removal of sialic acid [28]. In addition to this, NMR and dynamics studies on GD1b and GD1b-lactone showed deep conformational differences between the two molecules. In GD1b, the tetrasaccharide GalNAc-[Neu5Ac-Neu5Ac-]Gal has a circular arrangement leaving a highly hydrophobic region with several hydrogens pointing towards the center. The external Neu5Ac is close to the external Gal residue, and with its carboxyl group within Van der Waals contact with the OH group at position 9 of the lateral chain of the inner Neu5Ac. The Gal-GalNAc glycosidic linkage shows a high degree of freedom. In GD1b-lactone, the trisaccharide GalNAc-[Neu5Ac-]Gal forms a rigid, circular arrangement showing strong inter-residue contacts between proton 8 of inner Neu5Ac and both protons 1 and 5 of GalNAc. The lactonization of the disialosyl residue induces a strong variation of the preexisting torsional glycosidic angles φ and ω, leaving the external Neu5Ac far from the external Gal [29]. Thus, the interaction between gangliosides and proteins can be dramatically modified, due to dramatic changes of the ganglioside three-dimensional structure by the lactonization process.

GD1b-lactone can be formed by a specific enzyme or under mild acid pH from the parent ganglioside GD1b. GD1b was transformed into its lactone when added to cultured cerebellar granule cells that naturally have GD1b-lactone in their pattern, while cultured astrocytes, that do not have GD1b-lactone in their pattern, did not transform GD1b [30]. GD1b was also injected into the rat brain and the production of GD1b-lactone resulted to be time dependent [28]. These results together with the information on the increase of the brain GD1b-lactone along the human aging process [10] would suggest that the lactonization of GD1b is possibly governed by a specific enzyme. But at the same time, it seems that a specific acidic pH could also be responsible for the process. The ester formation requires the presence of protons. The lactonization of GD1b was studied at a ganglioside concentration between 1 µM and 1 mM in 1 µM − 3 mM HCl. Lactonization occurred very rapidly during the first hour and then proceeded slowly. The maximum rate of the lactonization reaction was achieved with H^+^-GD1b ratio < 1. The rate then decreased exponentially. At equilibrium, the ratio between GD1b and its lactone is 3:7. Similar experiments carried out on GM1 and GD1a did not allow to obtain the parent lactones, suggesting that the process of lactonization occurs primarily on the disialosyl chain [31].

**Fig. 5:**
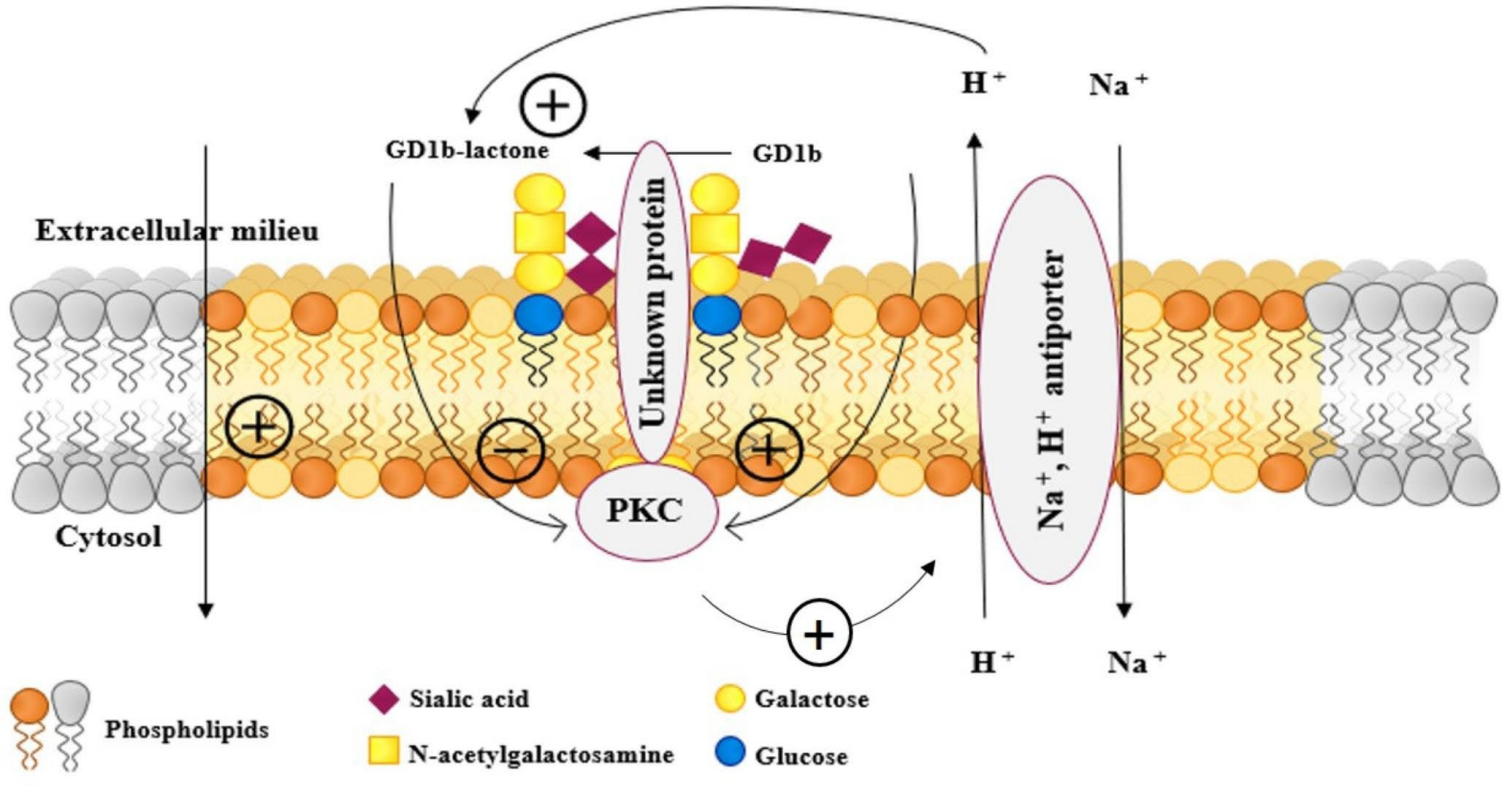
Proposed process involving the GD1b↔GD1b-lactone equilibrium for modulation of membrane kinase activity. GD1b-lactone formation is due to the acidic plasma membrane microenvironment. The catalytic protons are generated by the Na^+^/H^+^ pump located at the cell surface and activated through the action of protein kinase C (PKC). The local acidic microenvironment is directly responsible for lactone formation

Gangliosides are membrane components that are stably inserted into the outer layer. At the cell surface the pH is around 7 but we must recall that some specific proton pumps are associated with the same ganglioside microdomain in the brain [32]. These pumps are under control of kinases by phosphorylation and rapidly modify the pH in the ganglioside microenvironment by exchanging the external Na^+^ with the cytosolic H^+^. This is enough to modify the GD1b/GD1b-lactone equilibrium in favor of GD1b-lactone. On the other hand, GD1b is an activator of the phosphorylation process while GD1b-lactone is not [33]. Thus, the lactonization would block the acidification process, so that the Na^+^ returns to the outside of the cell and converts GD1b-lactone to GD1b. Figure 5 reports a cartoon of the above described possible process.

## Conclusion

The first information on the brain lipids goes back to the end of the nineteenth century thanks to the studies of J.L.W. Thudichum [34]. He introduced the word “ganglioside” for compounds present in human ganglions. To isolate and characterize gangliosides it was necessary to wait for more than an additional half of a century [35–37].

Gangliosides are components of all mammalian cells, being particularly abundant in the nervous system. Many ganglioside structures have been characterized and although it cannot be ruled out that very minor gangliosides are still to be isolated and characterized, it can be said that the large majority of cell patterns are known. Therefore, the main interest of scientists moved from ganglioside chemistry to ganglioside biochemistry. The information on the specific role played by gangliosides in modulating cell physiology is rapidly growing and the involvement of gangliosides in controlling the cell signaling processes is in many cases well understood [38–40]. Much is due to the interaction between the ganglioside and the extracellular portion of membrane proteins. The structure of the ganglioside oligosaccharide chain is the ganglioside portion that drives the interaction process. *O*-acetylation↔de-*O*-acetylation of sialic acid and ganglioside lactonization↔de-lactonization could be two processes developed by evolution to better regulate some aspects of the cell physiology. Investments on these two processes are necessary to understand their specific role. The particular complexity of this research represents interesting and challenging studies for scientists.
